# A polymer-based ratiometric intracellular glucose sensor†
†Electronic supplementary information (ESI) available: The synthesis of a sensor, sensor selectivity, and co-localizations. See DOI: 10.1039/c4cc01110d



**DOI:** 10.1039/c4cc01110d

**Published:** 2014-05-19

**Authors:** Liqiang Zhang, Fengyu Su, Sean Buizer, Xiangxing Kong, Fred Lee, Kevin Day, Yanqing Tian, Deirdre R. Meldrum

**Affiliations:** a Center for Biosignatures Discovery Automation , Biodesign Institute , Arizona State University , Tempe 85287-6501 , USA . Email: Yanqing.tian@asu.edu ; Fax: +1-480-727-6588 ; Tel: +1-480-965-9601

## Abstract

The glucose metabolism level reflects cell proliferative status. A polymeric glucose ratiometric sensor comprising poly(*N*-(2-hydroxypropyl)methacrylamide) (PHPMA) and poly[2-(methacryloyloxy)ethyl]trimethylammonium chloride (PMAETMA) was synthesized. Cellular internalization and glucose response of the polymer within HeLa cells were investigated.

## Abstract 2

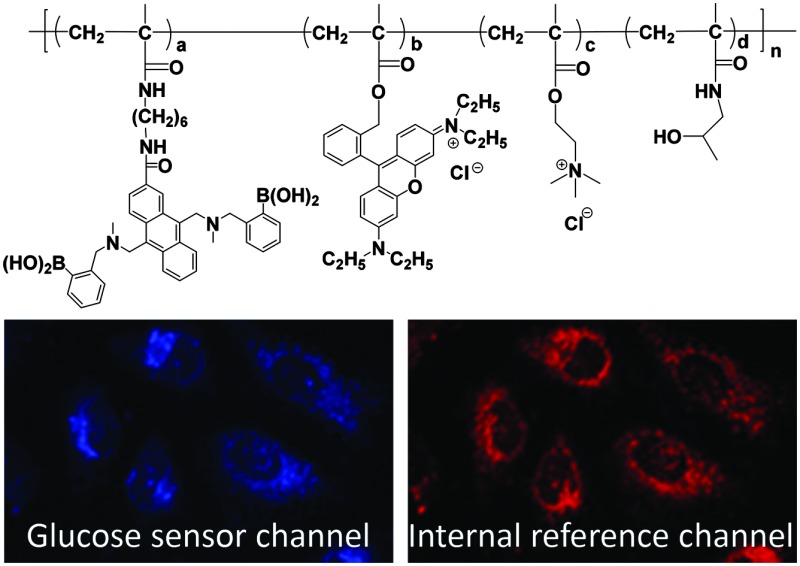
A new polymeric ratiometric glucose sensor was synthesized and used for dynamically monitoring intracellular glucose concentrations in HeLa cells.

Glucose is the major carbon donor and energy source for the maintenance of cell homeostasis and cell proliferation.^1,2^ Radioactive labeled glucose and the unmetabolized glucose analog (2-deoxyglucose) are commonly used for monitoring glucose uptake;^3–6^ however, these analogs are still different from glucose, with some of them inducing cell death.^7,8^ In order to monitor glucose uptakes and/or the changes of glucose concentrations, many different glucose sensors have been developed.^9^ Most of these sensors were designed to be able to detect the concentration changes of glucose in the environment of cells, *i.e.* extracellular glucose. A few kinds of glucose sensors were reported to be capable of real-time measurements of intracellular glucose concentrations, which include enzyme-based sensors,^10^ genetically encoded protein-based sensors,^11–13^ and the probes encapsulated by biologically localized embedding nanoparticles (PEBBLE).^14^

Our center has been working on developing fluorescent biosensors for understanding cell metabolism at bulk and single cell levels.^15–20^ Very recently, we have synthesized a polymer film-based extracellular dual sensor for glucose and oxygen.^21^ Herein, we report a polymeric sensor for intracellular glucose monitoring. The use of a cell permeable polymer platform as an intracellular sensor will enable the abundance of sensor materials and broaden the design flexibility of ratiometric sensors, which has not yet been explored. We use poly(*N*-(2-hydroxypropyl)methacrylamide) (PHPMA) (Scheme 1) as a biocompatible polymer, which has been widely used for drug delivery.^22^ The polymer possesses a small fraction of poly[2-(methacryloyloxy)ethyl]-trimethylammonium chloride (PMAETMA), which is a polymer with positive charges for ensuring its cell permeability.^20^ The glucose probe is a monomer, which can be easily copolymerized with HPMA and MAETMA. To alleviate the influence of a complex cellular environment on the accuracy of the measurement, a rhodamine-based glucose non-responsive probe (Rhod-MA) was integrated in the polymeric sensor for obtaining a ratiometric sensor.

**Scheme 1:**
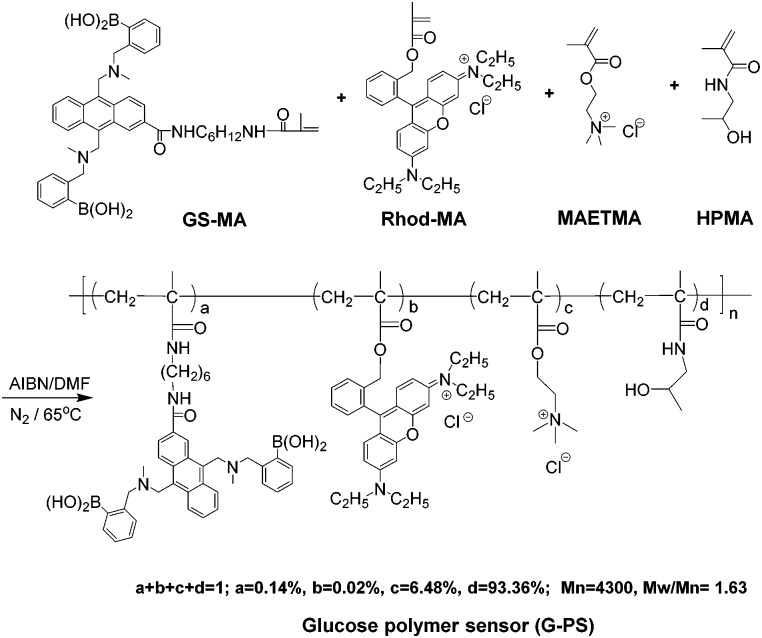
Synthesis of intracellular glucose sensor: G-PS. The ratios were determined by ^1^H NMR (Fig. S1 of ESI†) and UV-vis (Fig. S2, ESI†) spectra.

The sensor was synthesized using the traditional radical polymerization approach (Scheme 1) with the co-polymerization of Rhod-MA,^21^ GS-MA,^21^ HPMA^20^ and MAETMA in DMF. The polymeric glucose sensor (G-PS) was purified through precipitation from the DMF solution into acetone to remove any potential non-polymerized monomers and further by dialysis against water.

The sensor was characterized using gel permeation chromatography, and ^1^H NMR. Zeta potential measurement indicated the positive charge of the polymer with a zeta potential of 18.5 mV.

The response of the G-PS to glucose in phosphate buffer saline solution (PBS) is given in Fig. 1. The blue emission with a maximum at 445 nm increased with increasing glucose concentration. The fluorescence intensity at 445 nm is found to increase by about 4.5 fold with 50 mM of glucose as compared to that without glucose. This is due to the effect of photo-induced electron transfer (PET)^23^ (detailed mechanism of the PET effect on glucose sensing is given in ESI,† Fig. S3). The orange emission with a maximum at 580 nm from the rhodamine group did not show a response to glucose, indicating its suitableness as an internal built-in reference probe for the glucose sensor.

**Fig. 1:**
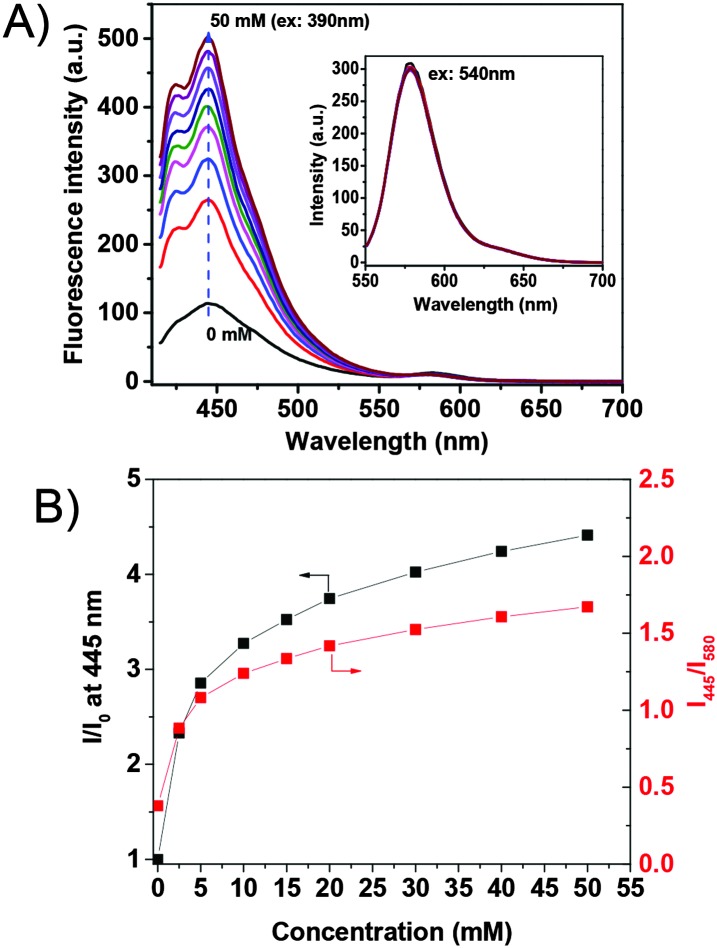
Typical responses of G-PS to glucose in PBS buffer. The inset figure in A shows the magnified peak at 580 nm under an excitation at 540 nm. (B) The glucose concentration dependent fluorescence intensity ratio changes. *I*_0_ is the fluorescence intensity at 445 nm before the interaction with glucose. *I* is the fluorescence intensity after interaction with glucose.

Fig. 1B shows plots of the intensity ratio changes with respect to glucose concentration. The sensor has excellent sensitivity to glucose concentration lower than 10 mM, especially in the concentration range from 0.1 to 5 mM. The sensor has linear response to glucose from 0.1 mM to 1 mM (Fig. S4, ESI†). Noting that the normal intracellular glucose concentration may vary from 0.1 to 5 mM depending on cell lines and status,^24,25^ we believe that this sensor is capable of monitoring intracellular glucose concentration.

The saccharide specificity of the sensor was compared among glucose, fructose, galactose and mannose. G-PS has responses to other saccharides and is most sensitive to fructose (Fig. S5, ESI†). This is common for many other amino-boronic-containing glucose sensors.^26^ Considering that there are few other saccharides except glucose used for cell culture, this specificity will not affect the sensor's application for the detection of glucose in cell metabolism research.

The sensor was internalized with human cervical cancer HeLa cell lines. We found that the sensor at a concentration of 0.05 mg mL^–1^ in cell culture medium could stain cells after 3 hours of cellular internalization. To get better cellular images, the sensor concentration of 0.1 mg mL^–1^ and an internalization time of 16 hours were usually used for cell staining. Results showed that the sensor is cell permeable, and localizes in the cytoplasm area. Fig. 2 shows the cellular distribution of the sensor in HeLa cells. The sensor is also cell permeable to other cell lines, like metaplastic epithelial CPA cells, glioblastoma U87-MG cells, and mouse macrophage J774.A1 cells (Fig. S6, ESI†). The blue color (Fig. 2A) represents the glucose probe, and the red color (Fig. 2B) represents the internal built-in probe. The pink color is the exact overlay of the images of Fig. 2A and B. It is worth noting that the ratio between the intensity of blue and red fluorescence of G-PS does not overlap well in some area of cells (Fig. 2C) which might be attributed to non-uniform subcellular distribution of glucose. The sensor's subcellular colocalization was further investigated using mitochondria-specific MitoTracker® Green and lysosome-specific LysoTracker® Green, respectively (Fig. S7 and S8, ESI†). Results showed no specific co-localizations of the sensor in the two important organelles. The possible influence of intracellular cellular pH on the sensor's responses to glucose was studied. The intracellular pH value was homogenized using a commercially available Intracellular pH Calibration Buffer Kit from pH 5.5 to 7.5 (Life Technology catalog number P35379) with valinomycin and nigericin, which helps equilibrate the pH inside and outside of cells. We did not find significant fluorescence changes from cellular pH 5.5 to 7.5 (Fig. S9, ESI†).

**Fig. 2:**
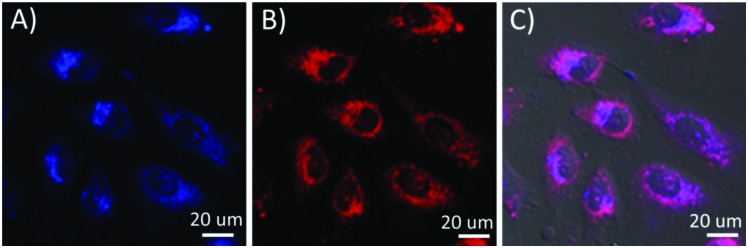
Cell images of G-PS in HeLa cells. (A) Blue channel for glucose probes excited at 405 nm; (B) red channel for rhodamine internal reference excited at 561 nm; (C) overlay of A and B with the bright field image.

The cytotoxicity of the sensor to HeLa cell lines was studied using 3-[4,5-dimethylthiazol-2-yl]-2,5-diphenyl tetrazolium bromide (MTT) assay (Fig. S10, ESI†). No significant cell cytotoxicity was observed at a sensor concentration of 0.05 mg mL^–1^ after internalization with cells for 24 hours.

The fluorescent response of G-PS to intracellular glucose changes was tested with HeLa cells. According to a known protocol,^6^ cells were treated by medium without serum for 16 hours before the glucose uptake experiments were performed in KRH buffer (50 mM of HEPES, 137 mM of NaCl, 4.7 mM of KCl, 1.85 mM of CaCl_2_, 1.3 mM of MgSO_4_ and 0.1% BSA). Intracellular glucose concentrations and their dynamic changes (Fig. 3) were determined by referring the titration curve. To check the influence of extracellular glucose concentration on intracellular glucose concentration, we used two extracellular glucose concentrations, *i.e.* 10 mM and 25 mM, respectively. It was found that the intracellular glucose concentration of the starved HeLa cells was 0.12 mM.^27^

**Fig. 3:**
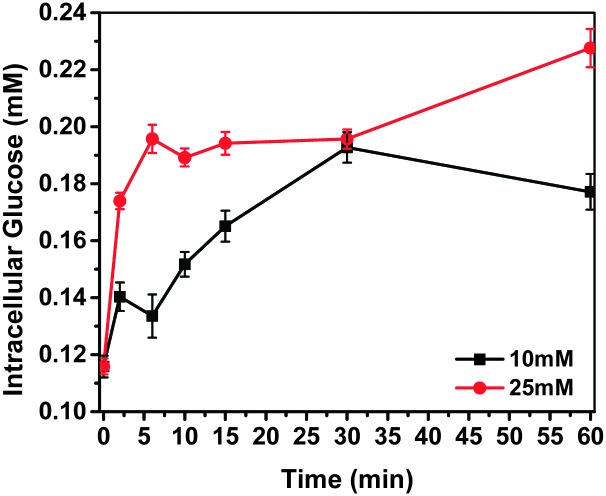
Intracellular glucose concentration detected by G-PS. 10 mM and 25 mM of extracellular glucose were applied to cell media after 60 min of glucose starvation.

After cells started to take up glucose from the KRH buffer, intracellular glucose concentration started to increase. At the high extracellular glucose concentration (25 mM), intracellular glucose reached equilibrium within 5 minutes. With an increase of incubation time, the glucose concentration did not change much. At the normal extracellular glucose (10 mM) concentration, it took about 30 minutes to reach the equilibrium of intracellular glucose. The intracellular glucose concentration after 30 minutes of internalization with glucose was determined to be 0.20 mM using the sensor G-PS.

In conclusion, we have developed a polymer-based ratiometric glucose sensor for monitoring the intracellular glucose concentration. It was demonstrated that the sensor is capable of measuring dynamic intracellular glucose concentration and the changes. Considering the flexibility of the sensor design using polymer technology, we believe that the use of polymers as intracellular sensors will broaden the design of sensors with multifunctionality. These polymer based sensors will provide a new and flexible platform for intracellular glucose sensing, and can be extended to other sensors. Further investigation of the use of this G-PS for single live cell glucose analysis is in progress.

This work was supported by the NIH National Human Genome Research Institute, Centers of Excellence in Genomic Science, grant number 5 P50 HG002360, and the NIH Common Fund LINCS program, grant number 5 U01 CA164250 (Professor Deirdre R. Meldrum, PI).
