# Supplementary material for: A polymer-based ratiometric intracellular glucose sensor
Source: Chem Commun (Camb). 2014 May 19;50(52):6920–2. doi: 10.1039/c4cc01110d (PMC4093769; doi:10.1039/c4cc01110d)
Supplement: Supplementary file 1 [file CC-050-C4CC01110D-s001.pdf]

Electronic Supplementary Material (ESI) for ChemComm.
This journal is © The Royal Society of Chemistry 2014

A polymer-based ratiometric intracellular glucose sensor

Liqiang Zhang, Fengyu Su, Sean Buizer, Xiangxing Kong, Fred Lee, Kevin Day, Yanqing Tian*, Deirdre R. Meldrum

Center for Biosignatures Discovery Automation, Biodesign Institute, Arizona State University, Tempe 85287-6501.

1. Materials and instruments

Chemicals including glucose, *N, N′*-dimethylformamide (DMF), azobisisobutyronitrile (AIBN), and [2-(methacryloyloxy)ethyl]-trimethylammonium chloride were commercially available from Sigma-Aldrich (St. Louis, MO) and were used without further purification. MitoTracker Green and LysoTracker Green were bought from Life Technologies (Grand Island, NY). Glucose probe (GS-MA) and the built-in internal reference probe (Rhod-MA) were prepared according to known procedures[1-2]. HPMA was synthesized according known procedure[3]. Doubly distilled water was used for the preparation of buffer solutions.

A Varian liquid-state NMR operated at 400 MHz for ^{1}H NMR was used for NMR spectra measurement. A Shimadzu RF-5301 spectrofluorophotometer was used for fluorescence measurements. Confocal microscope (Nikon, TE2000E, Melville, NY) was used for cell imaging. Plate reader (Biotek, Synergy H4, Winooski, VT) was used in the detection of glucose uptake. Zeatasizer Nano ZS (Malvern, United Kingdom) was used to measure the zeta potential of polymers.

2. Polymerization and characterization of the sensor

Synthesis of G-PS with feed molar ratios of a: b: c: d of 0.17: 0.03: 9.35: 90.45. 200 mg of HPMA, 30 mg of MAETMA, 2.0 mg of GS-MA[2], 2.0 mg of Rhod-MA[2] and 5.0 mg AIBN were dissolved in 3 mL of DMF. This solution was degassed three times through a standard freeze-thaw process. The monomers were polymerized at 65 °C for 16 hours under nitrogen. The polymer was precipitated into 150 mL of ether from the DMF solution. The pink polymer was re-dissolved in 3 mL methanol and re-precipitated into 100 mL of acetone. Yield: 160 mg (68.4%). Mn = 4300, Mw = 7000, Mw/Mn = 1.63. The incorporated molar ratios of each monomer in the polymer were determined using ^{1}H NMR and UV-visible spectra. The determined ratios are: 0.14: 0.02: 6.48: 93.36.

3. Experimental condition for cell culture, imaging, toxicity, and colocalization study

HeLa cells (American Type Culture Collection, ATCC, Manassas, VA) were cultured in Eagle's minimum essential medium (EMEM) supplemented with 10% fetal bovine serum, and incubated at 37 °C in 5% CO2 atmosphere. Cells were then seeded onto 96 well plates at 10,000 cells per well, and incubated for 1 day at 37 °C. The sensor G-PS dissolved in 10 mM HEPES buffer (pH 7.4) was added to the medium to make the final concentrations of the sensor in a range of 50 - 500 µg/ml. Three hours of internalization was found to be sufficient for achieving satisfactory images in the

colocalization assay. For the cell toxicity assay, cells were incubated with the sensor for 6, 12 and 24 hours before the assay was performed with the cytotoxicity MTT kit from Promega (CellTiter 96® Non-Radioactive Cell Proliferation Assay) following the detailed procedure from the company.

The glucose uptake assays were performed with the standard protocol[4]. A typical example was described below: Cells were incubated in serum free medium containing sensor (50 µg/mL) for 16 hours. Then cells were sequentially washed alternatively by PBS and KRH without glucose, followed by 30 min of incubation in KRH solution without glucose. The observations by plate reader were started immediately after the solution was changed to KRH with glucose (10 mM or 25 mM). Temperature was kept at 37 °C. 390 nm and 540 nm were applied as excitation wavelengths. Data was collected every 5 minutes.

4. Figures captions of the supplementary figures:

Figure S1: ^{1}H NMR spectrum of G-PS in D_2O.
Figure S2: UV-Vis spectrum of G-PS in H_2O (1mg/mL).
Figure S3: The photo-induced electron transfer (PET) mechanism of the glucose probe for sensing.
Figure S4: Glucose titration for the glucose sensor G-PS in KRH buffer with 0.1% BSA.
Figure S5: Selectivity of G-PS to a few saccharides.
Figure S6. Confocal images of Barrett's esophageal epithelial cells (A); Human glioblastoma-astrocytoma, epithelial-like U87-MG cells (B); and murine macrophage-like J774-A1 cells (C). a: blue channel for glucose probes; b: red channel for rhodamine reference probes; c: bright field images; d: overly of a, b, and c.
Figure S7: Colocalization study of G-PS with LysoTracker® Green for HeLa cells.
Figure S8: Colocalization study of G-PS with MitoTracker® Green for HeLa cells.
Figure S9: Influence of intracellular pH on the ratiometric fluorescence intensity changes. I_{445} is obtained from confocal microscope excited at 405 nm using the spectral model. I_{580} was obtained under confocal microscope excited at 561 nm. Intracellular pH value was homogenized using a commercially available Intracellular pH Calibration Buffer Kit from pH 5.5 to 7.5 (Life Technology catalog number P35379) with valinomycin and nigericin, which help equilibrate the pH inside and outside of cells.
Figure S10: Cytotoxicity of the sensor G-PS to HeLa cells.

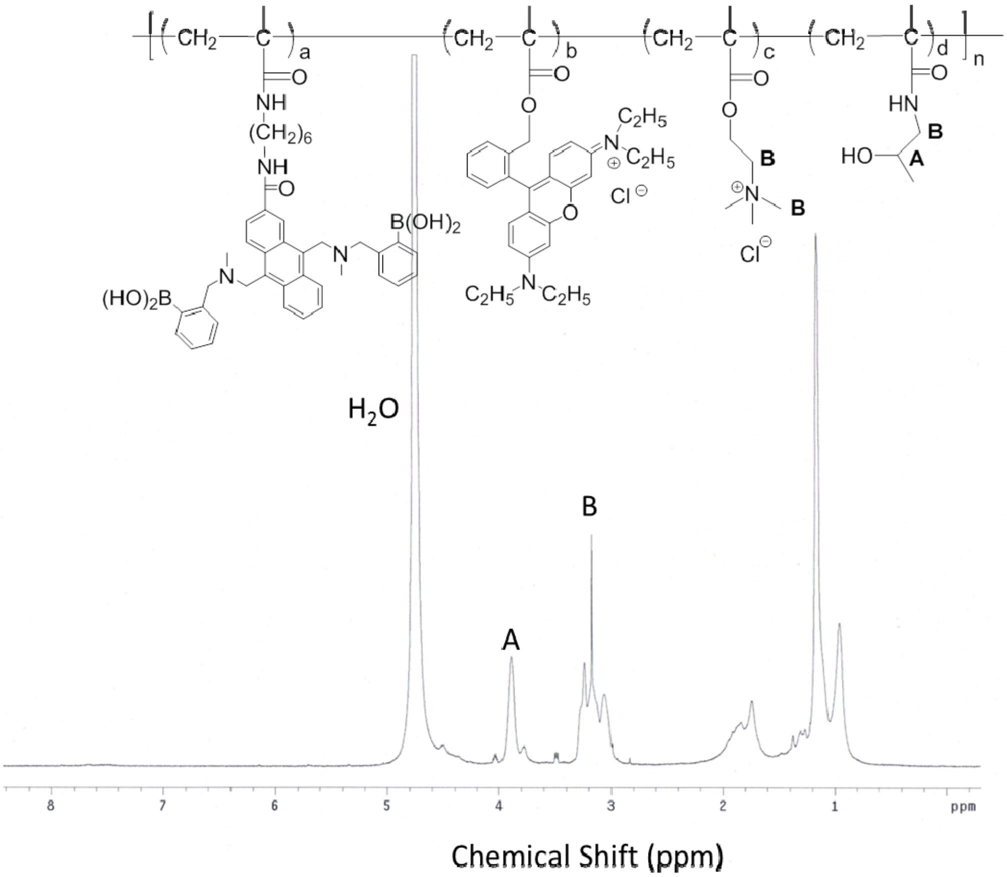

Figure S1. ^{1}H NMR spectrum of G-PS in D_2O.

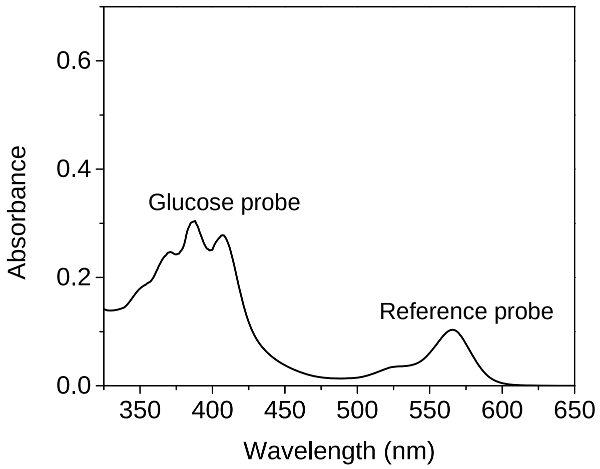

Figure S2. UV-Vis spectrum of G-PS in H_2O (1mg/mL).

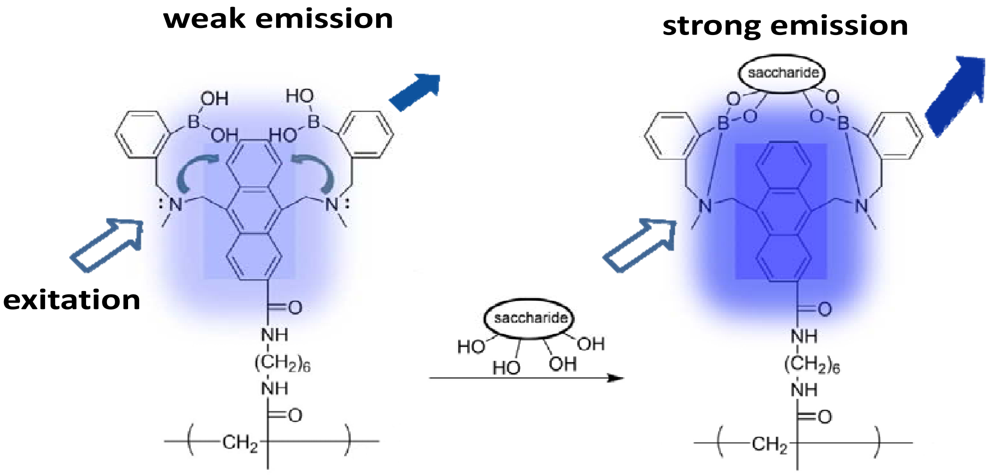

Figure S3: The photo-induced electron transfer (PET) mechanism of the glucose probe for sensing.

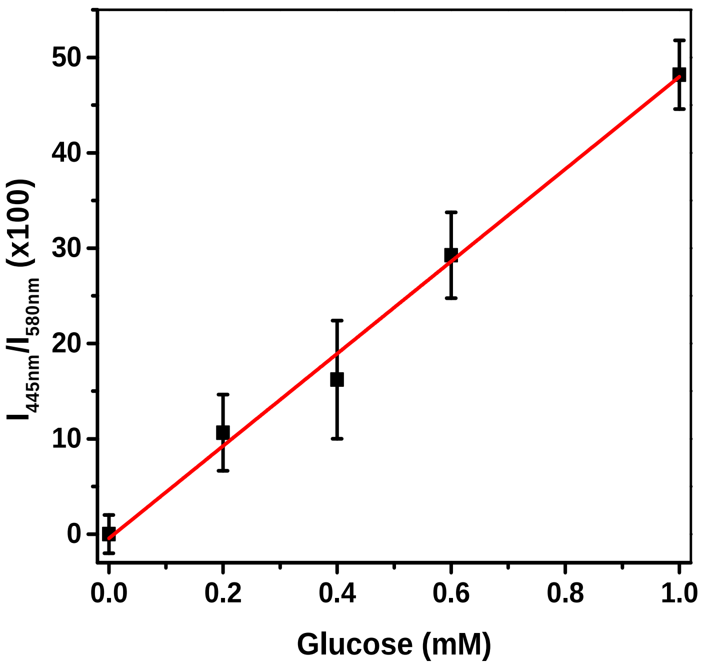

Figure S4: Glucose titration for the glucose sensor G-PS in KRH buffer with 0.1% BSA. G-PS concentration: 50 µg/ml.

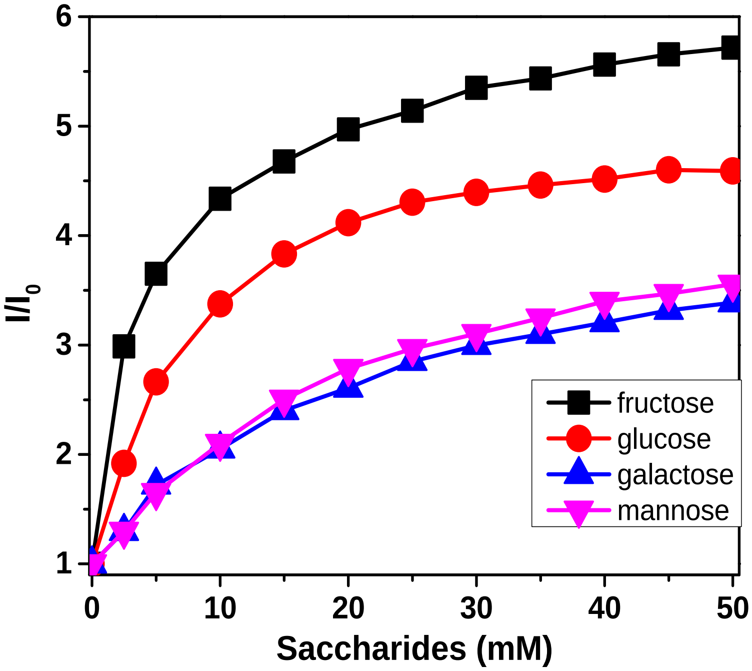

Figure S5: Selectivity of G-PS to a few saccharides. I_0 is the fluorescence intensity at 445 nm before the interaction with the saccharides. I is the fluorescence intensity after interaction with saccharides.

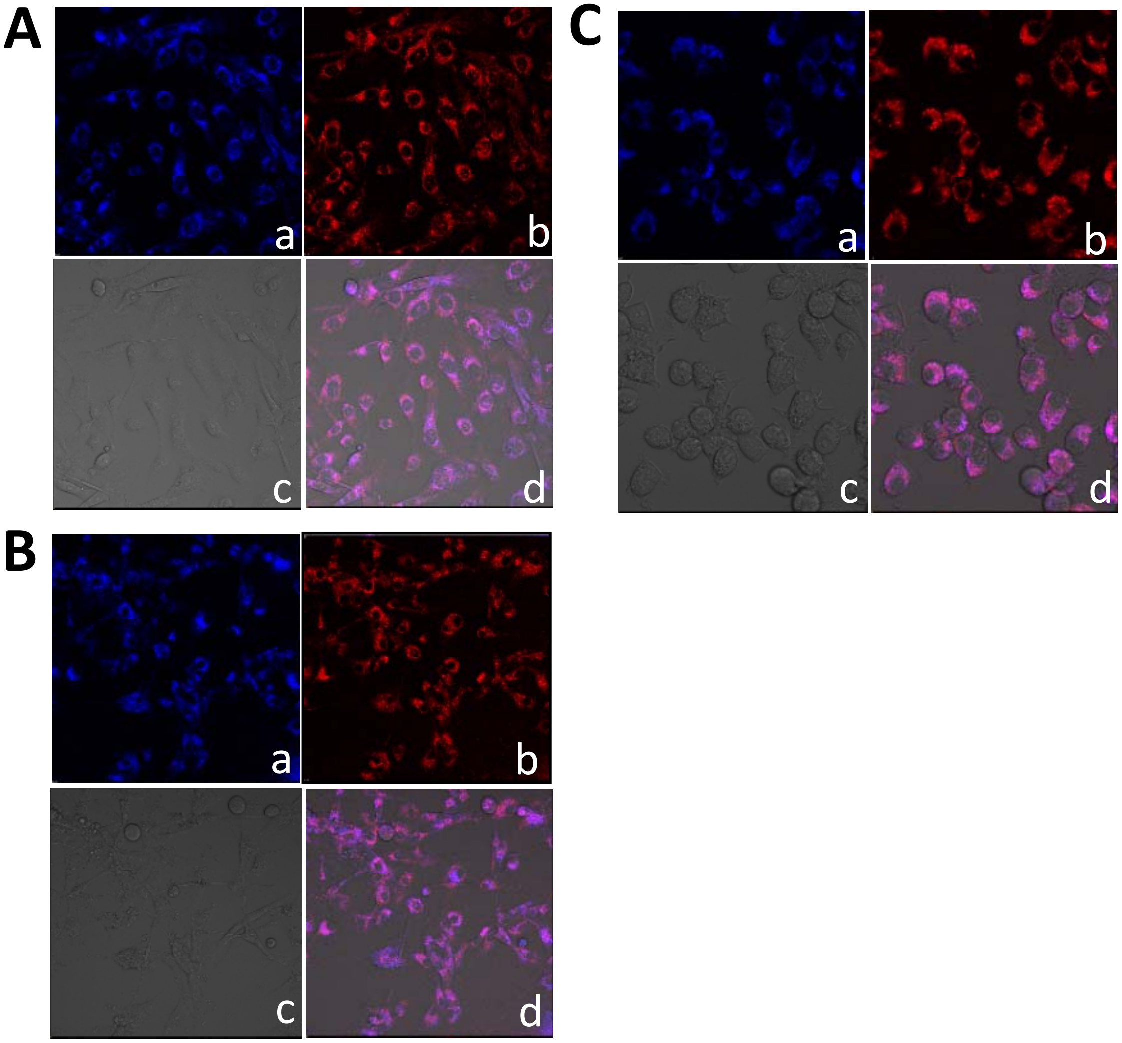

Figure S6. Confocal images of Barrett's esophageal epithelial cells (A); Human glioblastoma-astrocytoma, epithelial-like U87-MG cells (B); and murine macrophage-like J774-A1 cells (C). a: blue channel for glucose probes; b: red channel for rhodamine reference probes; c: bright field images; d: overly of a, b, and c.

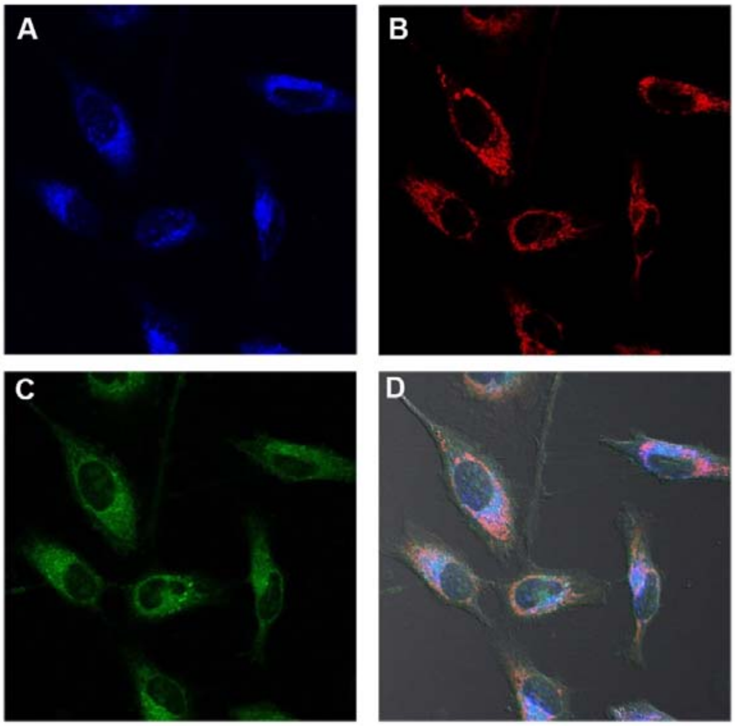

Figure S7: Colocalization study of G-PS with LysoTracker® Green for HeLa cells. 50 μg/mL of G-PS was used and the incubation time is 3 hours. 75 nM of LysoTracker® Green was used and the incubation time is 30 minutes. A) blue channel from the G-PS excited at 405 nm; B) red channel from the internal reference probe of rhodamine excited at 561 nm; C) green fluorescence from LysoTracker® Green excited at 488 nm; and D) the overlay of A, B, and C. The Pearson's correlation coefficient (Rr) of the blue and green channels is 0.62.

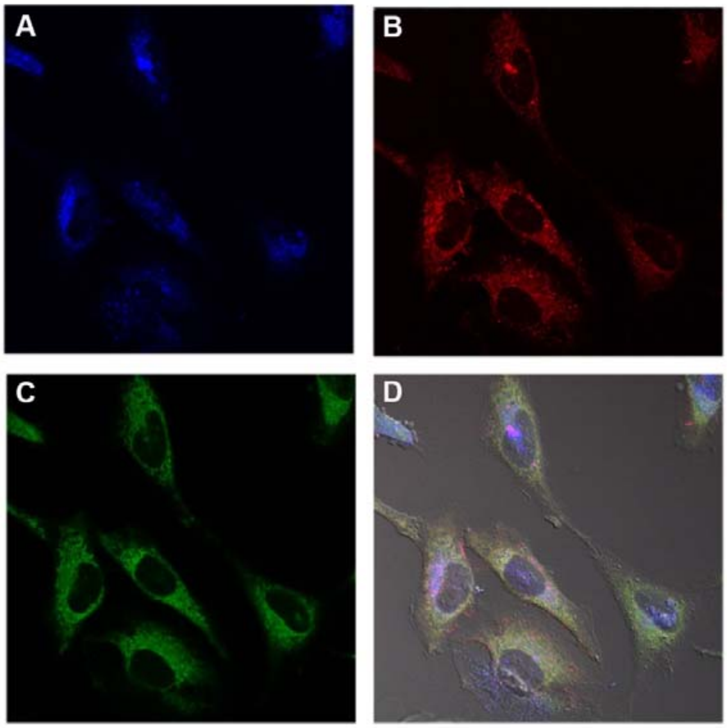

Figure S8: Colocalization study of G-PS with MitoTracker Green® for HeLa cells. 50 μg/mL of G-PS was used and the incubation time is 3 hours. 75 nM of MitoTracker Green® was used and the incubation time is 30 minutes. A) blue channel from the G-PS excited at 405 nm; B) red channel from the internal reference probe of rhodamine excited at 561 nm; C) green fluorescence from MitoTracker Green® excited at 488 nm; and D) the overlay of A, B, and C. The Pearson's correlation coefficient (Rr) of blue and green channels is 0.44.

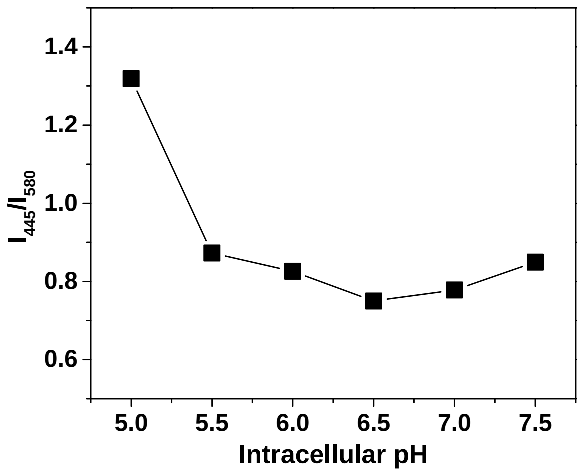

Figure S9: Influence of intracellular pH on the ratiometric fluorescence intensity changes. I_{445} is obtained from confocal microscope excited at 405 nm using the spectral model. I_{580} was obtained under confocal microscope excited at 561 nm. Intracellular pH value was homogenized using a commercially available Intracellular pH Calibration Buffer Kit from pH 5.5 to 7.5 (Life Technology catalog number P35379) with valinomycin and nigericin, which help equilibrate the pH inside and outside of cells.

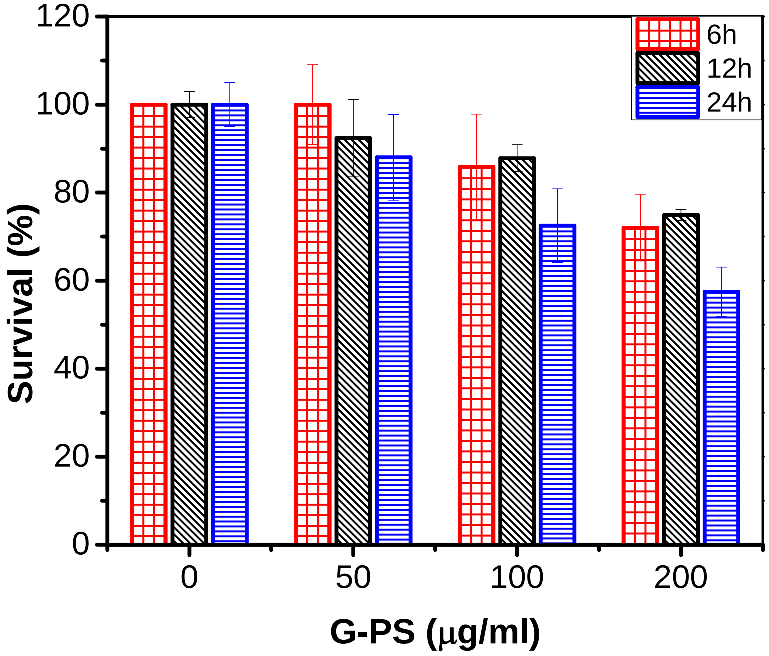

Figure S10: Cytotoxicity of the sensor G-PS to HeLa cells.

References:

1. Kawanishi, T.; Romey, M. A.; Zhu, P. C.; Holody, M. Z.; Shinkai, S. *J Fluoresc* **2004,** *14*, 499-512.
2. Zhang, L.; Su, F.; Buizer, S.; Lu, H.; Gao, W.; Tian, Y.; Meldrum, D. *Biomaterials* **2013,** *34*, 9779-88.
3. Lu, H. G.; Su, F. Y.; Mei, Q.; Tian, Y. Q.; Tian, W. J.; Johnson, R. H.; Meldrum, D. R. *J. Mater. Chem.* **2012,** *22*, 9890-9900.
4. Yamamoto, N.; Ueda, M.; Sato, T.; Kawasaki, K.; Sawada, K.; Kawabata, K.; Ashida, H. *Current protocols in pharmacology / editorial board, S.J. Enna* **2011,** *Chapter 12*, Unit 12 14 1-22.
